# Deformities of the Spine and Chest, Successfully Treated by Exercise Alone, and without Extension, Pressure, or Division of Muscles

**Published:** 1843-10

**Authors:** 


### Art. IV.

*Deformities of the Spine and Chest, successfully treated by Exercise alone, and without Extension, Pressure, or Division of Muscles.* By Chas. H. Rogers-Harrisson, m.r.c.s. &c. &c.—*London*, 1842. 8vo, pp. 164.

The title of Mr. Harrisson's work would seem to imply that it was merely intended to explain and recommend a particular plan of treatment in deformities of the spine and chest; but we learn from the "advertisement" that it is offered to the profession, and, by the way, to the non-medical world too, as a complete treatise on these affections. The following extract conveys, in the author's words, his own estimate of the nature of the book.

"My object has been to make this an easily intelligible, and, if possible, a useful book, both to the young practitioner and to the *enlightened general reader*. I have therefore generally *supposed very little to be previously understood, have not withheld even elementary details*, and have illustrated by simple lithographic outlines whatever might have been less intelligible without them. I have also drawn from all sources, English, French, and German, everything that I thought would render *the work tolerably complete*."

The italics in the above quotation are our own. We have introduced them to designate those passages which show that the work purports to be a complete treatise on deformities which constitute its subject; and that it is consequently a legitimate object of inquiry to examine how far this promise has been fulfilled.

Mr. Harrisson, in conformity with the plan above announced, commences with a description of the "normal structure of the parts liable to deformity;" and devotes forty pages, with eleven lithograph illustrations, to an account of the bones, ligaments, and muscles of the spine and thorax. In a work such as the present, we would be far indeed from desiring any laboured display of minute anatomical knowledge; but as the author has made his election to enter into anatomical details, we have a right to require that what he does say should be precise and accurate, and that, at the least, every point having a direct practical application should be clearly stated and its importance distinctly indicated; more particularly when the writer has "supposed very little to be previously understood," and premises that he has "not withheld even elementary details." But although a quarter of the entire book is occupied with anatomy, what is said on the subject is imperfect, inaccurate, and practically useless. We have neither sufficient time nor space at our command to point out the defects which we have noticed in this respect; but our readers may rely on finding our judgment confirmed by a perusal of Mr. Harrisson's volume. We must say a few words on the parts of the work having a more direct bearing on pathology and practice; and in doing so it will be our unpleasant duty to expose a remarkable instance of unacknowledged appropriation, on the part of the author, of another writer's labours.

The account of the slight lateral convexity to the right that naturally exists at the upper part of the dorsal portion of the spine, is singularly inaccurate and confused. At page 8 it is said to exist "sometimes,"

while at page 85 we are told it exists "early in *all* persons." As to its cause, Mr. Harrisson unhesitatingly adopts Bichat's theory, that it is due to the predominant action of the right arm. But he quotes Bichat (p. 80) as if he attributed the morbid and not the natural lateral curvature to this cause. At page 8 we are told that Cheselden erroneously believed this natural deviation to be designed "to make more room for the heart." We do not mean to advocate Cheselden's opinion, but as Mr. Harrisson noticed it, he should have adverted to the intimate relation, pointed out by Serres, that exists between the position of the heart at the left side of the spine and the usual direction of the dorsal curvature; at all events it is a curious coincidence that in preternatural lateral deviations of the spine, the heart and the liver, in the vast majority of cases, correspond to the concavity of the primary and secondary curvatures, which must to a certain extent tend to prevent their functions being disturbed. At page 53, Mr. Harrisson seems to misapprehend one of the theories that have been advanced on this subject: the author is there considering the causes of *morbid* lateral curvature, and says, "the extreme frequency of this curvature has even made many anatomists believe that it is the effect of some natural disposition, and they have attributed it to the presence of the arch of the aorta which beats against the left side of the anterior part of the bodies of most of these vertebræ, which by deviating from their natural direction commonly constitute it." This is certainly as strange a misconception as we recollect to have met with, unless indeed the inaccuracy is to be set down to mere slovenliness of expression. No anatomist ever attributed *morbid* lateral curvature of the spine (of which Mr. Harrisson is most distinctly speaking in the passage quoted,) to the pulsation of the arch of the aorta; many have, indeed, accounted for the great frequency of the *disease* by the existence of its *rudiment* as a *natural* disposition; and the existence of this rudiment some have referred to the presence of the arch of the aorta. Mr. Harrisson seems to misunderstand, and certainly ridicules, the theory of these anatomists. If their theory was what he represents it to be, it would certainly be absurd enough; but the influence of the presence of the arch of the aorta in producing the *natural* curvature so often mentioned, though perhaps much exaggerated, should scarcely perhaps be altogether rejected, when we know that in some instances of transposition of the viscera this curvature has been to the left, as was observed by M. Grisolles, for example, in two cases; and when we further recollect, as Cruveilhier has expressly remarked, that this is possibly but a particular instance of the general law, that bones present a depression whenever a large artery is in their immediate vicinity. The reader may perhaps suppose that we have dwelt on this point too long, but it is an average example of the confused and disconnected way in which Mr. Harrisson treats every subject he touches; and besides this was a matter which he should have discussed in the fullest and clearest manner, as he adopts Bichat's view respecting the generation of this natural deviation of the spinal column, and extending the same principle to morbid deviations, makes it one of the main foundations of his theory of the etiology and treatment of the affection.

It is very difficult to discover what Mr. Harrisson's opinion respecting the etiology of lateral curvature of the spine really is (p. 43.) He attributes the affection to a derangement of the equilibrium of muscular power, saying, "In fact we attribute these deformities to a want of equality of power which ought to have existed between the muscles of the back having attachment to various points of the spinal column, and exerting an action on the body." But at pages 50-1, the muscles which thus cause the deformity are said to be only altered in consequence of that deformity, from "the effects of such a change in the structure of the bony framework to which they are attached. Those whose insertion is *at a distance* must naturally be distended and elongated," while "the muscles whose attachments *are near* are, on the contrary, reduced to a state of *relaxation* which tends to contract and shorten them." Strongly confused as is this paragraph, we may yet guess at what the author intends to convey. The *distance* or *proximity* of the attachments of the muscles, of course, has nothing to say to the matter; but we suppose Mr. Harrisson's meaning to be, that the muscles which correspond to the convexity of the curvature are distended, while those on the side of the concavity are *relaxed*, and if they be relaxed of course they could have no influence in producing the deformity. At page 74, however, we are distinctly told that "the inequality of the muscular power of the spine is *rarely primitive*, but appears to be *almost always* owing to a continued position of that part in the same vicious direction." The illustration of this proposition is amusing. "A child whose *spine is deformed*, in standing upright for some time, generally does not keep its feet in the same line, but one is placed before the other. Curvature, however temporary, is the result." (p. 75.) That is to say, a certain deformity produces a certain position, and this position produces the deformity of which it is the result, in other words, a cause is produced by its effect. The author afterwards reverts to this original position, and again maintains that inequality of muscular action is the predominant and most frequent cause of lateral curvature of the spine.

"It must then be evident to all who do not decide upon the cause of any abnormal state without taking into account the physiological phenomena which attend its development, that this curvature is, *in almost all cases*, the result of an increase in the vital energy, and, consequently, in the physical development, which the muscles of the left spinal hollow and of the right shoulder acquire, from the habit early taught to children, of making use much more frequently of the right than of the left hand." (pp. 87-8.)

But though the preponderant use of the right arm is thus stated to be the cause of the deformity in "almost all cases," great importance is attached to the malign influence of "that circular instrument of constriction applied to the chest, and known under the name of stays," (p. 56,) and to a bad carriage or faulty attitude unduly persevered in, (p. 72.) Mr. Harrisson employs twelve pages in expounding the ills that flow from the use of tight stays, and strongly objects to them not only as a physician but as a man of taste.

We are far indeed from undervaluing the injurious effects of tight-lacing, or of a faulty attitude, and still further, of course, from blaming Mr. Harrisson for having noticed and dwelt on those points, which have an intimate and important connexion with his subject; but we do blame the loose, vague, inconsistent and contradictory statements which he makes respecting the causes of spinal deformity.

Mr. Harrisson, we have seen, refers almost all cases of lateral curvature of the spine to the custom of using the right hand more frequently than the left. In explaining how this habit produces the effect in question, he adopts Pelletan's theory without the slightest acknowledgment, and, not content with this, borrows the very language in which it is conveyed.

Before we proceed to establish this charge of plagiarism we must premise that Pelletan's views are published in his *Réflexions sur les Causes qui determinent la Courbure de la Colonne vertébrale*, (Journal de Maisonnale, t. ii, p. 372,) which we have not an opportunity of consulting, but an abstract of his theory is given by M. Malgaigne, (Anat. Chirurg, t. ii, pp. 18-19). Whether Mr. Harrisson has copied directly from Pelletan's memoir we do not know, but certain it is that the entire passage which we shall immediately quote from M. Malgaigne appears in Mr. Harrisson's book translated almost literally, though not inserted exactly in its original shape, as its continuity is broken up by the intercalation of numerous sentences and several entire paragraphs. We do not know whether the superadded matter exists in Pelletan's original memoir or whether it has been inserted by Mr. Harrisson himself. The only difference, however, that this makes is, that in the former case Mr Harrisson has borrowed more than we have been able to detect, in the latter he has taken no small pains to conceal his obligation to M. Malgaigne, and we should rather hope that the former of these two alternatives may prove the correct one. The following are the passages from M. Malgaigne, and their unacknowledged translation by Mr. Harrisson; the preceding observations sufficiently explain the broken shape in which the latter extract appears.

"Toutes les fois qu'une partie du corps doit exécuter des mouvemens sur son ensemble, la première condition est la fixité des points auxquels s'insèrent les muscles qui vont la mouvoir. Le bras ne saurait agir par ceux de ses muscles qui s'attachent à l'omoplate, que l'omoplate ne soit solidement fixée, et elle ne peut l'être qu'autant que ses muscles propres, le trapèze, le rhomboïde, l'angulaire, le grand dentelé, trouvent eux-même un point fixe ailleurs; ce point fixe pour les trois premiers est la colonne vertébrale.

"Chez un sujet à muscles forts, à ligamens serrés, les muscles propres à la colonne suffisent pour la maintenir droite et stable; un sujet faible n'y arrive pas toujours, mais il obtient cette stabilité d'une autre manière. Ainsi le rachis dans la rectitude est un corps élastique et flexible que le moindre effort peut entrainer dans une direction quelconque. Mais si cette tige vient à être courbée, elle offre tout-à-coup une rigidité proportionnelle à l'éntendue de sa courbure et qui résulte d'un antagonisme entre sa force élastique qui tend à la redresser et la puissance qui la courbe. Voyez le tireur en garde dans l'escrime, il a le rachis fortement courbé à guache; l'enfant qui s'essaie à ouvrier de la main droite une porte qui résiste est contourné en arc presque demi-circulaire, etc. Ils courbent la colonne pour lui donner plus de fixité.

"Or cette fixité nécessaire aux mouvemens énergiques ne l'est pas moins à des mouvemens légers, mais qui demandent beaucoup de précision. La jeune personne qui brode au plumetis, qui dessine ou qui écrit, doit se premunir contre la vaccillation du rachis qui entraînerait celle de l'épaule et de la main; elle le tient donc légèrement courbé dans sa partie supérieure; cette flexuosité, qu'on regarde généralement comme une habitude vicieuse, n'est autre chose que la condition indispensable pour un sujet faible de l'exécution de certains actes du bras droit. Aussi est-il très souvent impossible, malgré les remonstrances les plus fréquentes, d'obtenir de la jeune fille la plus docile cette rectitude tant désirée des mères de famille." (Malgaigne, Traité d'Anat. Chirurg., etc., t. ii, pp. 18-9.)

"Every time any part of the body has to execute a motion, the first condition of the motion is to fix the points to which the muscles which have to move it are attached . . . . . In the motions of the thoracic extremity the shoulder must be fixed . . . . . The shoulder-blade, which is to present to the motions of the arm the fixed points which are requisite for them. . can be rendered immoveable only by the simultaneous contraction of the trapezoid, rhomboid, etc. . . but it becomes evident that the shoulder-blade itself cannot be fixed in an immoveable manner, unless the superior part of the vertebral column, and the head present in their turn a fixed point for the trapezoid,* *angular*, rhomboid, etc.

"In an individual well made and in the enjoyment of all her muscular vigor the habitual efforts of the superior extremities may be carried on in the manner just spoken of; but in one whose muscular powers are extremely reduced, the muscles of the neck........may not be sufficient to give a proper stability to the upper part of the dorsal region, and nature........gives to this delicate being means of another kind........The spinal column, when upright, is an elastic and flexible body, which the least effort may put into any direction whatever...... .But if a straight elastic pillar can be made to bend by the slightest effort, this same elastic pillar........offers at once a rigidity which is proportioned to the extent of the curvature, and which is the result of an opposition between the elastic power which tends to straighten it and the power which curves it........The fencing-master, on guard, has his spinal column forcibly bent to the left side; the child who attempts to open a door with the right hand is bent to almost a semi-circle.

" We must further remark, that the precision and exactness of certain delicate movements require as much the upper portion of the spinal column to be fixed, as certain abrupt and powerful motions. A young person embroidering at a frame, drawing, or writing, is obliged to guard against the vacillation of the spinal column, which would produce that of the arm and hand; and consequently preserves it curved in its upper part, however slightly........We now see that the bending of the spinal column in the superior part of the dorsal region........ instead of being the effect of a vicious habit, is no more than an indispensable condition for the execution of certain motions of the right arm, so that........it is often quite impossible, in spite of the most frequently repeated care and remonstrances, to obtain from the most docile and submissive young person that erectness so desired by mothers." (Harrisson on Deformities of the Spine, &c., pp. 80-4.)

We thus find that *fourteen* disconnected fragments, scattered through *five* pages, constitute, when the intervening passages are omitted, a continuous, close, and, for the most part, literal translation of the passages quoted from M. Malgaigne's work! We leave it for Mr. Harrisson to explain and account for this strange circumstance: we have felt it our duty to expose the fact thus openly. We took care to quote at the commencement of this article Mr. Harrisson's general acknowledgment of having drawn freely from all sources whatever he thought would render his book tolerably complete; but we cannot regard this as in the least justifying the appropriation of the precise ideas and language of other authors, whose names are never mentioned: we cannot even regard the general confession as an extenuation of the particular offence; indeed, it might easily be made to appear an aggravation of it. Mr. Harrisson himself evidently cannot regard the general acknowledgment as sufficient, for he in several instances mentions by name authors whom he quotes.

Our remaining observations on the present work must be brief. The anatomical characters of the parts engaged in lateral curvature of the spine are scarcely noticed. We are indeed told, at page 42, that

* The French name retained in the translation.

the intervertebral fibro-cartilages and the bodies of the vertebræ are diminished in thickness on one side; but it is erroneously added, "on the contrary, the other side increases in thickness." It is not stated that the cartilages are absorbed to a much greater extent than the bones; nor is there any allusion to the change that is effected in the symmetry of *every part* of the two sides of the affected vertebræ. The curious and most important fact, that the vertebræ, when lateral curvature has existed for any length of time, undergo a kind of rotation or torsion, is indeed mentioned under the head of "accessory deviations" (p. 92); but the nature of the alteration is most imperfectly given. M. Pelletan's explanation of the mode in which this torsion occurs is here again quietly adopted without his name being mentioned, and the French text is literally translated without interpolation or alteration! The reader, however, is not informed of the objections that have been made to this explanation; that several others have been proposed; and that the question has a direct and important bearing on the treatment of lateral curvatures of the spine,—M. J. Guerin's method of *sigmoid extension*, for example, being based, in part at least, on this view (as we think erroneous) of the mechanism by which this torsion is effected.

We shall not enter into any lengthened consideration of the mode of treatment recommended by Mr. Harrisson. He is vehement, even to vituperation, against the employment of all mechanical means; maintains that those who adopt them are "guided rather by the love of gain, than the interest of science and the love of public good," (p. 109;) and that "they have not furnished one single well-authenticated instance of cure." (p. 110.) But he admits that they have sufficient intellect to have "been capable of observing that the best means of refreshment, after long fatigue, is repose." (p. 109.) Now, we freely admit that the employment of mechanism in the treatment of distortions of the spine has been monstrously abused; that it has been and is a fruitful source of charlatanism and the vilest quackery; and is daily applied in numerous cases without any the slightest necessity. We are further well aware that the application of mechanical means has often done more harm than good; is not unfrequently inefficient; and when it does good is too often tediously slow in effects. All this we admit, insomuch so that we are firmly persuaded the best mode of treating considerable lateral curvatures of the spine is a problem yet to be solved; but it is something more than absurd to maintain that mechanical means have never effected "one single well-authenticated instance of cure;" and it is, at the least, indecent to brand, "as being solely guided by the love of gain," the many conscientious practitioners who have employed them.

Mr. Harrisson recommends what he terms the "rational treatment," which means attention to position and certain gymnastic exercises. Mr. Harrisson even claims some originality on this score; he admits that

"The medical men most skilled in this department have, indeed, in their writings and in their practice inculcated the necessity of gymnastics in the general treatment of some of these diseases........but they only provided for a secondary requisite; let us provide for the primary one; they have only opposed the progress of the disease; let us endeavour to point out the true means of curing it in a very great majority of cases." (p. 127.)

And again,

"I hope by assigning to the different muscles of which the vertebral column supports the action, the share which each may have in the production of curvature, and by reducing all to *geometrical data* to be able to prove that if gymnastic exercises, which, in the greater number of cases, ought to form the basis of the treatment of this deformity, especially at the commencement, have not always been productive of good effect, it is because they have not been prescribed in a distinct manner; and few have pointed out the particular kind of exercise required by each species of deviation." (pp. 128-9.)

We have in vain looked for any reduction of the matter to *geometrical data*, or for one tittle that is new in the directions for the application of gymnastic exercises. With respect to lateral curvature, the whole of the *geometrical data* and of the promised precision consist in sixteen lithograph sketches (with explanatory letter-press) of young ladies performing "the military extension motions," "the British military club exercise," and the "Indian club exercise." There is no notice of the equilibrium exercises recommended by Delpech, and which are perhaps the only ones admissible when the curvature is advanced; and the inefficacy and occasional prejudicial consequences of the exercises that are recommended, when the curvature is considerable, are not adverted to.

---

